# Utilization of antiretroviral therapy services and associated factors among adolescents living with HIV in northern Uganda: A cross-sectional study

**DOI:** 10.1371/journal.pone.0288410

**Published:** 2023-07-13

**Authors:** Innocent Odongo, Barbara Arim, Patrick Ayer, Tom Murungi, Susan Akullo, Docus Aceng, Henry Oboke, Edward Kumakech, Celestino Obua, Anna Grace Auma, Richard Nyeko

**Affiliations:** 1 Department of Public Health, Lira University, Lira, Uganda; 2 Department of Nursing and Midwifery, Lira University, Lira, Uganda; 3 Department of Community Psychology, Lira University, Lira, Uganda; 4 Department of Pharmacology and Therapeutics, Mbarara University of Science and Technology, Mbarara, Uganda; 5 Department of Paediatrics and Child Health, Lira University, Lira, Uganda; Zimbabwe Health Interventions, ZIMBABWE

## Abstract

**Background:**

Suboptimal utilization of antiretroviral therapy (ART) services remains a problem among adolescents in low- and middle-income countries, which has a negative impact on their response to treatment and increases the risk of developing resistance. Optimal use is essential to enhancing treatment efficacy. We investigated the optimal use of ART service and predictors among adolescents living with HIV (ALHIV) in northern Uganda.

**Methods:**

We used a cross-sectional study design to collect quantitative data from 293 ALHIV at three health facilities in Lira municipality, northern Uganda. We used an interviewer-administered questionnaire and data abstraction form. Data were analysed using SPSS version 23 software. Descriptive analysis and logistic regressions were performed to determine the relationship between the predictor and outcome variables. Statistical significance was determined at P-value<0.05 and the adjusted odds ratio with a 95% confidence interval was used.

**Results:**

The level of utilization of ART services was suboptimal among 27.6% (81/293) of the participants, and only 63.1% (185/293) were virally suppressed. Of the participants who were optimally utilizing ART services, the majority 86.8% (184/212) were virally suppressed. Age 10–14 years (aOR = 3.34), the presence of both parents (aOR = 1.85), parental and peer reminders (aOR = 2.91) and (aOR = 0.49) respectively, and being on ART for five years or less were the characteristics related with optimal utilization of ART services.

**Conclusions and recommendations:**

Not all ALHIV used ART services to their full potential. However, factors such as participants’ age, the presence of both parents, reminders from parents and peers, and being on ART for some time were all related to the optimal use of ART services. There is a need for developing strategies to increase family and peer support with a focus on older adolescents if the 95-95-95 goal is to be achieved in this age group.

## Introduction

On a global, regional, and national scale, HIV continues to disproportionately affect adolescents [1], defined by the World Health Organization (WHO) as those between the ages of 10 and 19 years [2]. Recent estimates indicate that 410,000 [196,000–650,000] young people aged 10 to 24 years were newly infected with HIV in 2021 alone, 150,000 [44,000–310,000] of whom were adolescents aged 10 and 19 years [3]. In Uganda, the Joint United Nations Programme on HIV/AIDS (UNAIDS) estimated that 1.4 million people were living with HIV by 2020, more than 110,000 of whom were adolescents and young adults [4].

The successful scale-up and effectiveness of ART have resulted in an increasing number of children living with HIV surviving and developing into adolescence [5]. Maintaining optimal use of ART services by adolescents living with HIV (ALHIV) has become a significant healthcare concern for this group as the number of adolescents receiving ART rises [6]. Evidence from sub-Saharan African nations shows that ALHIV have significantly greater attrition rates, both before and after the start of ART [7, 8]. This may compromise the goals of ART [9] and have detrimental effects on viral suppression [10, 11], stopping transmission [12], and preventing antimicrobial resistance in environments with few resources [13].

The Ugandan government has made several attempts to increase the populace’s access to and use of HIV services, with a particular focus on high-risk groups, including adolescents. However, the ART and care indicators for adolescents fall far short of the desired targets, with 67.4% being virally suppressed compared to the national viral load suppression (VLS)target of 92.2% [14], even though the country is reported to be on track to achieving the UNAIDS’s 95-95-95 targets. The country is reported to have already met the second 95 target and approaching the third 95 target for VLS) [14].The UNAIDS’s 95-95-95 targets call for 95% of people living with HIV (PLHIV) to be aware of their HIV status, 95% of those aware of their status to be on ART, and 95% of those on ART to achieve VLS.

Maximizing the utilization of ART services is vital to meet the UNAIDS’s 95-95-95 targets among this unique adolescent population. However, there is a paucity of information on how adolescents in northern Uganda make optimal use of ART services, underscoring the need for greater understanding in this area to develop evidence-based interventions to ensure HIV-positive adolescents in low-resource settings optimally use ART services. While earlier studies were conducted on ALHIV, the majority of those studies concentrated on adherence to ART [15–17], with little attention paid to the level and factors influencing the usage of ART services in general. Additionally, the majority of the previous studies were conducted among adolescents in different socio-economic contexts from the current study setting. The purpose of this study was to describe the utilization of ART services and the associated factors among the ALHIV in northern Uganda.

## Methods

### Study design and setting

We used a cross-sectional study design to collect quantitative data in August 2020. The study area was Lira Municipality, Uganda. Lira Municipality is located in northern Uganda at a distance of 338 kilometres from Kampala, the capital city of Uganda. Adolescents aged 10–19 years make up about 27% of the 99,392 population of Lira Municipality. The study was carried out in three public health facilities: Ober Health Centre III, four kilometres in the western part of Lira Municipality; Ayago Health Centre III, three kilometres in the eastern part of Lira Municipality; and Lira Regional Referral Hospital (LRRH), located within Lira City Centre. A health centre III in Uganda, which is situated at the sub-county geopolitical administrative level, serves a population of approximately 20,000 people and offers basic curative and preventive services, including HIV/AIDS treatment, care, and support. Although Lira municipality has a population of 99,392 as of the National Population and Housing Census 2014, the Regional Referral Hospital (LRRH) provides general and speciality treatments to over 2.3 million residents in the nine districts that make up the Lango sub-region of Uganda. The study area (Lira Municipality)was selected because it has one of the highest HIV prevalence rates in Uganda compared to the national average (7.6% vs. 5.5%), and one of the lowest viral load suppression rates overall (67.4%) and among adolescents and young adults (50.7%) which are lower than both the national averages and the UNAIDS’s 95-95-95 targets [14].

### Study population

The study was conducted among ALHIV aged 10–19 years from Lira Municipality receiving ART services at the three public health facilities (Ober Health Centre III, Ayago Health Centre III, and Lira Regional Referral Hospital), who have been in care for at least one year.

### Sample size estimation

A sample size of 293 participants was estimated using the single population proportion formula by Kish Leslie [18] based on a proportion (P) of 74.4% [19], with a marginal error (D) of 5%, a score in the standard normal curve (Z) corresponding to 95% certainty (1.96).

### Data collection procedure

We used an interviewer-administered questionnaire to collect data in August 2020. The tool was developed based on the study objectives. We pre-tested the data collection tool, outside our study area, among 10 HIV-positive adolescents attending the ART clinic at Amach Health Centre IV, located in Erute South, Lira District. The results of this testing were used to improve the tool: related questions were merged, poorly understood statements were rephrased, and wrongly spelled words were corrected, among others. The improvement of the tool involved clinical experts in HIV care to assure its content and face validity. The calculation for the internal consistency (i.e., Cronbach’s alpha) of the tool was not applicable since the measure of the outcome variable was just one item, not a scale. The questionnaire collected information on three areas: the participant’s socio-demographic characteristics, family and social support systems, and health system factors. Further data were extracted from the unit ART registers on clinical variables such as ART initiation, compliance with the ART services scheduled appointment, and viral load, among others, covering a period of ten years, from August 2010 to July 2020.

The data were collected by undergraduate students of public health, midwifery and community psychology, following their training in the data collection procedures. The study sites were purposively selected because of their patient load and wide catchment areas, while systematic random sampling was used to select the participants at each of the facilities. The participants were systematically randomly sampled on each clinic day after being consecutively arranged based on their registration numbers. According to a preliminary survey of records from the health facilities, it was estimated that, on average, about 19 adolescents attend the hospital on a daily basis, while around 9 attend health centers III (HCIIIs). As a result, we recruited nine participants from the hospital each day, as well as four from each of the two health centers III. Consequently, every second adolescent (19/9 for the hospital and 9/4 for the HCIIIs) was chosen for the study, with the first participant chosen at random from the allocated numbers. The first participant was chosen at random, and then every other participant from the list was selected, and so on, until the proportionate number of participants from each facility was reached. The data were then collected from the participants after obtaining their written informed consent by signature or thumbprint.

### Outcome variables

The outcome variable was the proportion of the ALHIV who made optimal use of the ART services. We defined ≥95% compliance to the scheduled visits for ART-related services as optimal use of the ART services. The socio-demographic characteristics of the participants, the family and social support systems, and aspects of the health system were considered among the potential predictor variables.

### Data management and statistical analysis

We assured the quality of the data during the collection process by checking for the completeness of the questionnaire and medical record data extraction form at the end of each day and addressing all identified gaps to ensure completeness and consistency. Data were entered, cleaned, and analyzed using the Statistical Package for Social Sciences (SPSS) software package (SPSS for Windows, Version 23.0, Chicago, SPSS Inc.). Descriptive statistics were used to summarize categorical variables as proportions and continuous variables as means (standard deviation) and median (interquartile range). The Chi-square test (for categorical variables) with odds ratios and 95% confidence intervals were used to examine the association between the potential predictors and the outcome variable. The multivariate logistic regression model was used to assess the factors that independently predicted optimal utilization of ART services and was reported as an adjusted odds ratio (aOR) at a 95% confidence level. P-values of 0.05 were considered statistically significant. The multivariate model included variables that were statistically significant at the bivariate level (p 0.05) and those with p values of 0.2 but made scientific senses (biological or social) to be reconsidered as potential predictors. The data underlying the findings in this manuscript is available as S1 Dataset.

### Ethical considerations

The study was approved by the Gulu University Research and Ethics Committee (GUREC-050-20) and registered with the Uganda National Council of Science and Technology (RESCLEAR/01). Additional administrative approvals for this study were obtained from the Lira district’s chief administrative officer, district health officer, hospital administrator, and the in-charges of the health facilities. Before data collection, voluntary written informed consent was obtained from adolescents aged 18–19 years, the ones who were married (emancipated minors), or the parents/guardians of those aged 10–17. Before participation in the study, participants aged 10–17 assented to participate in the study. Confidentiality was maintained at all stages by using codes in place of participants’ names.

## Results

### Description of the study population

**Fig 1:**
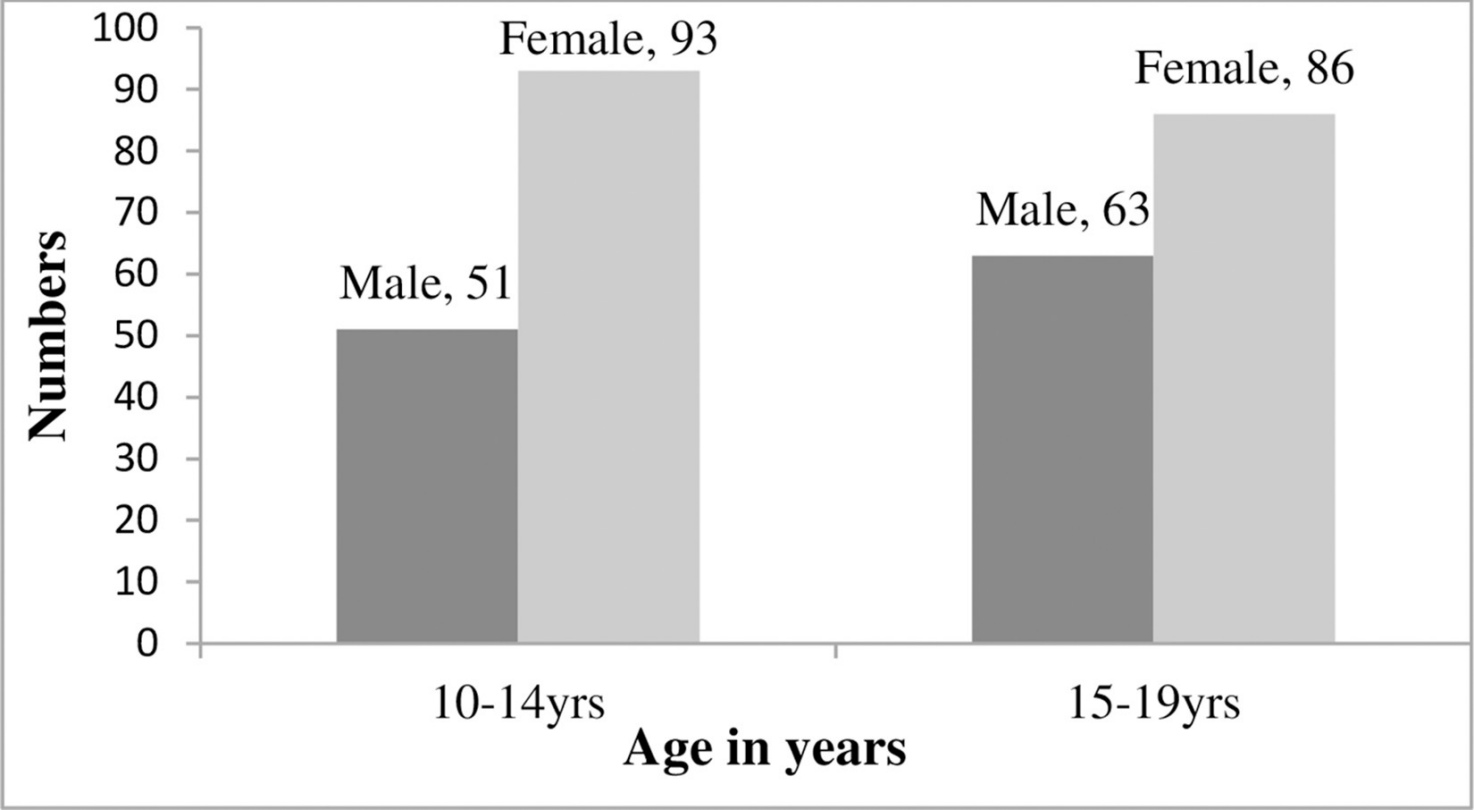
Age-sex distribution of the study population.

A total of two hundred ninety-three (293) ALHIV with a median age of 15.0 years (IQR 13.0–17.0) from the study settings participated in the study (Table 1). Female adolescents made up 61.1% (179/293) of the respondents, and they likewise dominated both the younger (10–14 years) and older (15–19 years) adolescent age groups (Fig 1). More than half of the respondents, 56.7% (166/293), had been on ART for five years or fewer, and nearly two-thirds, 71.3% (209/293), had only completed their primary level education or had no formal education at all. Only 40.6% (119/293) of the individuals had both of their parents still alive, and less than half, or 41.6% (122/293), had disclosed their HIV status to their parents and other family members.

**Table 1:** Socio-demographic characteristics of adolescents living with HIV in northern Uganda in August 2020 (n = 293).

Characteristic		n	%
**Age (years)**	Median 15.0 (IQR 13.0–17.0)		
10–14		144	49.1
15–19		149	50.9
**Sex**			
Male		114	38.9
Female		179	61.1
**Education level**			
≤Primary		209	71.3
Post-primary		84	28.7
**Occupation**			
Employed		35	11.9
Not employed		258	88.1
**Marital status**			
Married		17	5.8
Not married		276	94.2
**Duration on ART (years)**	Median 5.0 (IQR 4.0–7.5); Range 8 (3–11)		
≤5 year		166	56.7
More than 5 years		127	43.3
**Disclosure of HIV status**			
Disclosed		122	41.6
Not disclosed		171	58.4
**Parental living status**			
Both alive		119	40.6
One or both died		174	59.4

IQR: Inter-quartile range

### The utilization of ART services and virologic suppression among ALHIV in northern Uganda

In the study population, 72.4% (212/293) of the adolescents living with HIV utilized ART services at an optimal level (Table 2). There was a positive correlation between the level of utilization of ART services and viral load suppression (VLS) (p = 0.01). In total, 86.8% (184/212) of adolescents who used ART services optimally were virally suppressed at the time of the study, compared to only 1.2% (1/81) of those who used ART services sub-optimally. Overall, only 63.1% (185/293) of the study participants were virally suppressed. Up to 99.3% (291/293) of the participants had a favourable opinion of the HIV care and treatment services offered at the sites.

**Table 2:** Summary of the utilization of ART services and virologic suppression among adolescents living with HIV in northern Uganda, August 2020 (n = 293).

Characteristic	n	%
**Optimally utilized**	**212**	**72.4**
Virally suppressed	184	86.8
Not virally suppressed	28	13.2
**Not optimally utilized**	**81**	**27.6**
Virally suppressed	1	1.2
Not virally suppressed	80	98.8

### Factors associated with optimal utilization of ART services among ALHIV in northern Uganda

When compared to their peers aged 15–19, the younger adolescents living with HIV aged 10–14 years old were 3.3 times more likely to make optimal use of ART services, and this association remained statistically significant at multivariate analysis (p <0.001, 95% CI 1.85–6.02) (Table 3). Adolescents who did not have any form of employment (literally no personal source of income) were noticeably less likely to make optimal use of the ART services when compared to their counterparts who were employed (p = 0.027, 95% CI 0.20–0.91). Further analysis revealed that all the adolescents employed were aged at least 16 years, the legal age of employment. Optimal use of the ART services was twice as likely to be attained by participants who had primary level education or no formal education at all, p = 0.006 (OR 2.17, 95% CI 1.26–3.74), but this did not independently predict optimal use at multivariate analysis (p = 0.452).

**Table 3:** Factors associated with optimal use of the ART services among adolescents living with HIV in Lira Municipality northern Uganda, August 2020 (n = 293).

Variable	Bivariate			Multivariate		
	OR	95% CI	*P*-value	aOR	95% CI	*P*-value
**Age (years)**						
10–14	3.95	2.24–6.96	**<0.001**	3.34	1.85–6.02	**<0.001**
15–19		1			1	
**Sex**						
Male	1.20	0.71–2.04	0.500		-	
Female		1				
**Education level**						
Primary/none	2.17	1.26–3.74	**0.005**	1.31	0.65–2.62	0.452
Post-primary		1			1	
**Occupation**						
Employed	0.27	0.13–0.55	**>0.001**	0.43	0.20–0.91	**0.027**
Not employed		1			1	
**Marital status**						
Not married	1.91	0.70–5.20	0.199		-	
Married		1				
**Duration on ART (years)**						
≤5§	2.13	1.26–3.57	**0.004**	1.80	1.02–3.19	**0.044**
>5		1			1	
**Disclosure status**						
Not disclosed	0.60	0.36–1.01	0.054		-	
Disclosed		1				
**Parental living status**						
Both are alive	2.26	1.29–3.96	**0.004**	1.85	1.03–3.34	**0.040**
One/both dead		1			1	
**Number of siblings**						
≤3	0.46	0.27–0.78	**0.004**	0.45	0.26–0.78	**0.005**
>3		1			1	
**Parental support**						
Yes	2.61	1.23–5.51	**0.010**	0.90	0.36–2.27	0.819
No		1			1	
**Parental reminders**						
Yes	3.09	1.75–5.46	**<0.001**	2.91	1.60–5.30	**<0.001**
No		1			1	
**Peer reminders**						
No	0.53	0.32–0.90	**0.017**	0.49	0.29–0.86	**0.012**
Yes		1			1	
**Drug side effects**						
Yes	0.78	0.42–1.42	0.411		-	
No		1				
**Perceived pill burden**						
Yes	1.23	0.47–3.20	0.670		-	
No		1				
**Opportunistic infection**						
Yes	0.08	0.04–0.17	**<0.001**	0.09	0.04–0.19	**<0.001**
No		1			1	

aOR: adjusted Odds Ration; §based on the median duration of ART

Adolescents with both living parents were 1.8 times more likely to make optimal use of ART services than those with either one or both deceased parents (p = 0.040, 95% CI 1.03–3.34). Similar to this, adolescents who received parental reminders to attend their ART clinic appointments were about three times more likely to make optimal use of those services than their counterparts who did not (p0.0001, 95% CI 1.60–5.30). On the other hand, adolescents with four or more siblings and those who did not receive reminders from their peers were significantly less likely to utilize the ART services optimally than their counterparts who had three or fewer siblings, p = 0.005 (OR 0.45; 95% CI 0.26–0.78) or those who were reminded by their peers, p = 0.012 (OR 0.49; 95% CI 0.29–0.86), respectively.

Adolescents on ART for five or fewer years were 1.8 times more likely than those on ART for much longer to make optimal use of the ART services (p = 0.044, 95% CI 1.02–3.19). However, compared to their peers who did not suffer episodes of opportunistic infections, adolescents who got episodes of opportunistic infections were less likely to optimally utilize the ART services (p<0.0001, 95% CI 0.04–0.19).

## Discussion

The findings in this study show that there is poor utilization of ART services among adolescents living with HIV in northern Uganda. As a result, of the expected 95% viral suppression rate [1] we found a viral suppression rate of only 63.1%. Other studies among adolescents have shown equally low viral suppression rates. By attending to ≥95% of the scheduled appointment visits at the time the study was conducted, which is how this study defined optimal ART service use for ALHIV, we were able to show that this proportion was low. Age, parental status, peer and parental support, and the length of ART were the main variables linked to the optimal use of ART services. The almost none existent virologic suppression among teenagers who sub-optimally used ART services compared to those who did so optimally is noteworthy, and of concern, if the 95-95-95 targets are to be realized. Antiretroviral therapy (ART) has been successfully scaled up, and as a result, more children living with HIV are surviving and developing into adolescence. As such, there is a need for continued support for these teenagers to stay in care and maximize the benefits of ART [5].

We also found that the percentage of adolescents living with HIV who disclosed their status was extremely low, a finding consistent with those reported in other studies [20–22], and that adolescents who had not disclosed their HIV status were less likely to optimally utilize ART services. Other reports show that adolescents must disclose their HIV status [15–17], as disclosure and adherence to care and treatment go hand in hand. Disclosure creates opportunities for adolescents to access adherence support and other forms of psychosocial support from family members, peers, and healthcare providers [23, 24].

### Factors associated with optimal utilization of ART services among ALHIV in Northern Uganda

According to our study, the best use of ART services was among younger adolescents. Adolescents aged 10 to 14 were thrice more likely than those aged 15 to 19 to optimally use ART services. An observation by Nachega et al. in South Africa that older adolescents living with HIV were less adherent and had lower rates of virologic suppression and immunological recovery [25] is supported by the subpar optimization of ART services among older adolescents in our study. Our finding also resonates with that of Mustapha et al, who found that only 19.7% of adolescent mothers aged 15–19 in Mulago Hospital, Uganda, optimally utilized the services for PMTCT [26]. The apparent independence and lack of consideration shown to older teenagers in both family and community settings may be one explanation for this finding.

Whether the parents were still alive or not had a substantial impact on the adolescents with regard to optimal usage of ART services. When compared to adolescents who had one or both of their parents deceased, those who had both of their parents alive were substantially more likely to use ART services to their full potential. Similar results were reported by Xu L et al. in Thailand, who found that adolescents who were not being cared for by their biological parents were more likely to be non-adherent [27]. This result supports the notion that family structure, support, and involvement are critical to the success of HIV care and treatment for adolescents. Encouragement, counselling, financial assistance, and paying close attention to their needs are some ways to achieve this [28, 29].

In our study, the significance of peer and family support was further emphasized concerning the adolescents’ motivation through repeated reminders and the optimization of ART service consumption. When compared to their peers who did not receive parental reminders, adolescents in this study who received reminders to attend their ART clinic services were nearly three times more likely to make the best use of those services. Similarly, adolescents who did not receive reminders from their peers were much less likely than their counterparts to make the best use of ART services. Our results may not come as a surprise given how important peer and emotional support are in motivating adolescents to utilize HIV services [23, 26, 28]. Peers and family support can help adolescents stick with their treatment plans because it eases the burden of having a disease, fosters optimism, lessens depressive symptoms and enhances healthy behaviour [23, 30, 31].

The strong correlation between optimal ART service utilization and ART duration was another important finding from our study. The median time on ART in this study was 5 years (IQR: 4.0–7.5), and adolescents on ART for five years or fewer were more likely to use their ART services to their full potential than those who had been on ART for a significantly longer duration. This result is surprising since one would think that someone who has been on ART for a longer period would be an expert client who understands and values the services and advantages of adherence to planned service appointments. However, as suggested by research by other authors, one potential explanation for this phenomenon can be related to the idea of treatment fatigue, demonstrating the negative effect of the life-long nature of ART on optimal adherence [28, 32, 33]. The difficulties older adolescents face transitioning from paediatric to adult ART therapy may also be the cause of low perceived readiness to transition care [34], with the potential for suboptimal service utilization.

Adolescents who suffered episodes of opportunistic infections (OIs) were less likely to use ART services optimally than their counterparts who did not experience episodes of OIs. This finding is consistent with that reported in a systematic review and meta-analysis by Abebe et al., where the presence of one or more OIs was associated with an increased risk of patient attrition [35].Temporally, this relationship can be two-sided in that, on the one hand, the occurrence of OIs can be a result of suboptimal ART utilization with the attendant poor virological suppression, and on the other hand, it can be a cause for non-adherence and interruption of ART service utilization. The latter conjecture was evidenced in a finding by Tegegne et al., where feeling sick was referenced, among other reasons for patients’ non-adherence [36].This study, however, did not investigate the temporal association between the occurrence of OIs and suboptimal utilization of ART.

### Study limitation

The study was conducted in only three health facilities within Lira municipality and hence the findings may not necessarily be generalizable to the whole region or other contexts. However, the facilities selected are high-volume facilities with wide catchment areas, making the findings externally valid to a good extent. The use of Odd ratios (ORs) from logistic regression instead of the relative risk ratio from the Poisson regression carried the risk of overestimating the association between the predictors and the outcome.

### Conclusions and recommendations

Not all adolescents living with HIV used ART services to their full potential. However, factors such as participants’ age, the presence of both parents, reminders from parents and peers, and being on ART for some time were all related to the optimal use of ART services. There is a need for developing strategies to increase family and peer support with a focus on older adolescents if the 95-95-95 goal is to be achieved in this age group.

## Supporting information

S1 Checklist STROBE checklist. (PDF)

S1 Dataset (XLSX)
