# Supplementary material for: Utilization of antiretroviral therapy services and associated factors among adolescents living with HIV in northern Uganda: A cross-sectional study
Source: PLoS One. 2023 Jul 13;18(7):e0288410. doi: 10.1371/journal.pone.0288410 (PMC10343037; doi:10.1371/journal.pone.0288410)
Supplement: S1 Checklist — (PDF) [file pone.0288410.s001.pdf]

STROBE Statement—checklist of items that should be included in reports of observational studies

	Item No.	Recommendation	Page No.	Relevant text from manuscript
**Title and abstract**	1	(*a*) Indicate the study's design with a commonly used term in the title or the abstract	1	We used a cross-sectional study design to collect quantitative data
		(*b*) Provide in the abstract an informative and balanced summary of what was done and what was found	2	We used an interviewer-administered questionnaire and data abstraction form. Data were analysed using SPSS version 23 software. Descriptive analysis and logistic regressions were performed to determine the relationship between the predictor and outcome variables. The level of utilization of ART services was suboptimal among 27.6% (81/293) of the participants, and only 63.1% (185/293) were virally suppressed. Of the participants who were optimally utilizing ART services, the majority 86.8% (184/212) were virally suppressed. Age 10-14 years (aOR = 3.34), the presence of both parents (aOR = 1.85), parental and peer reminders (aOR = 2.91) and (aOR=0.49) respectively, and being on ART

				for five years or less were the characteristics related with optimal utilization of ART services.
**Introduction**				
Background/rationale	2	Explain the scientific background and rationale for the investigation being reported	3	The successful scale-up and effectiveness of ART have resulted in an increasing number of children living with HIV surviving and developing into adolescence. Maintaining optimal use of ART services by adolescents living with HIV (ALHIV) has become a significant healthcare concern for this group as the number of adolescents receiving ART rises. Evidence from sub-Saharan African nations shows that ALHIV have significantly greater attrition rates, both before and after the start of ART and lower rates of retention in care.
Objectives	3	State specific objectives, including any prespecified hypotheses	4	The purpose of this study was to describe the utilization of ART services and the associated factors among the ALHIV in northern Uganda.
**Methods**				

Study design	4	Present key elements of study design early in the paper	4	We used a cross-sectional study design to collect quantitative data
Setting	5	Describe the setting, locations, and relevant dates, including periods of recruitment, exposure, follow-up, and data collection	4	The study area was Lira Municipality, Uganda. Lira Municipality is located in northern Uganda at a distance of 338 kilometres from Kampala, the capital city of Uganda. Adolescents aged 10-19 years make up about 27% of the 99,392 population of Lira Municipality.
Participants	6	(*a*) *Cohort study*—Give the eligibility criteria, and the sources and methods of selection of participants. Describe methods of follow-up<br>*Case-control study*—Give the eligibility criteria, and the sources and methods of case ascertainment and control selection. Give the rationale for the choice of cases and controls<br>*Cross-sectional study*—Give the eligibility criteria, and the sources and methods of selection of participants	N/A	
		(*b*)*Cohort study*—For matched studies, give matching criteria and number of exposed and unexposed<br>*Case-control study*—For matched studies, give matching criteria and the number of controls per case	N/A	
Variables	7	Clearly define all outcomes, exposures, predictors, potential confounders, and effect modifiers. Give diagnostic criteria, if applicable	6	The outcome variable was the proportion of the ALHIV who made optimal use of the ART services. We defined ≥95% compliance to the scheduled visits for ART-related services as optimal use of the ART services

Data sources/measurement	8*	For each variable of interest, give sources of data and details of methods of assessment (measurement). Describe comparability of assessment methods if there is more than one group	*N/A*	
Bias	9	Describe any efforts to address potential sources of bias	N/A	
Study size	10	Explain how the study size was arrived at	5	A sample size of 293 participants was estimated using the single population proportion formula by Kish Leslie based on a proportion (P) of 74.4%, with a marginal error (D) of 5%, a score in the standard normal curve(Z) corresponding to 95% certainty (1.96).

Continued on next page

Quantitative variables	11	Explain how quantitative variables were handled in the analyses. If applicable, describe which groupings were chosen and why	7	Descriptive statistics were used to summarize categorical variables as proportions and continuous variables as means (standard deviation) and median (interquartile range). The Chi-square test (for categorical variables) with odds ratios and 95% confidence intervals were used to examine the association between the potential predictors and the outcome variable.
Statistical methods	12	(*a*) Describe all statistical methods, including those used to control for confounding	7	We assured the quality of the data during the collection process by checking for the completeness of the questionnaire and medical record data extraction form at the end of each day and addressing all identified gaps to ensure completeness and consistency. Data were entered, cleaned, and analyzed using the Statistical Package for Social Sciences (SPSS) software package (SPSS for Windows, Version 23.0, Chicago, SPSS Inc.). Descriptive statistics were used to summarize categorical variables as proportions and continuous variables as means (standard deviation) and median (interquartile range). The Chi-square test (for categorical variables) with odds

				ratios and 95% confidence intervals were used to examine the association between the potential predictors and the outcome variable. The multivariate logistic regression model was used to assess the factors that independently predicted optimal utilization of ART services and was reported as an adjusted odds ratio (aOR) at a 95% confidence level. P-values of 0.05 were considered statistically significant. The multivariate model included variables that were statistically significant at the bivariate level (p 0.05) and those with p values of 0.2 but made scientific senses (biological or social) to be reconsidered as potential predictors.
		(*b*) Describe any methods used to examine subgroups and interactions	N/A	
		(*c*) Explain how missing data were addressed	N/A	
		(*d*) *Cohort study*—If applicable, explain how loss to follow-up was addressed<br>*Case-control study*—If applicable, explain how matching of cases and controls was addressed<br>*Cross-sectional study*—If applicable, describe analytical methods taking account of sampling strategy	N/A	
		(*e*) Describe any sensitivity analyses	N/A	
Results				
Participants	13*	(a) Report numbers of individuals at each stage of study—eg numbers potentially eligible, examined for eligibility, confirmed eligible, included in the study, completing follow-up, and analysed	8	A total of two hundred ninety-three (293) ALHIV with a median age of 15.0 years (IQR

				13.0–17.0) from the study settings participated in the study
		(b) Give reasons for non-participation at each stage	N/A	
		(c) Consider use of a flow diagram		
Descriptive data	14*	(a) Give characteristics of study participants (eg demographic, clinical, social) and information on exposures and potential confounders	8-9	A total of two hundred ninety-three (293) ALHIV with a median age of 15.0 years (IQR 13.0–17.0) from the study settings participated in the study (Table 1). Female adolescents made up 61.1% (179/293) of the respondents, and they likewise dominated both the younger (10–14 years) and older (15–19 years) adolescent age groups (Fig 1). More than half of the respondents, 56.7% (166/293), had been on ART for five years or fewer, and nearly two-thirds, 71.3% (209/293), had only completed their primary level education or had no formal education at all.
		(b) Indicate number of participants with missing data for each variable of interest	N/A	
		(c) *Cohort study*—Summarise follow-up time (eg, average and total amount)		
Outcome data	15*	*Cohort study*—Report numbers of outcome events or summary measures over time		
		Case-control study—Report numbers in each exposure category, or summary measures of exposure		
		Cross-sectional study—Report numbers of outcome events or summary measures	9-10	In the study population, 72.4% (212/293) of the adolescents living with HIV utilized ART services at an optimal level

Main results	16	(*a*) Give unadjusted estimates and, if applicable, confounder-adjusted estimates and their precision (eg, 95% confidence interval). Make clear which confounders were adjusted for and why they were included	10-12	When compared to their peers aged 15–19, the younger adolescents living with HIV aged 10–14 years old were 3.3 times more likely to make optimal use of ART services, and this association remained statistically significant at multivariate analysis (p <0.001, 95% CI 1.85–6.02) (Table 3). Adolescents who did not have any form of employment (literally no personal source of income) were noticeably less likely to make optimal use of the ART services when compared to their counterparts who were employed (p = 0.027, 95% CI 0.20-0.91). Optimal use of the ART services was twice as likely to be attained by participants who had primary level education or no formal education at all, p = 0.006 (OR 2.17, 95% CI 1.26-3.74), but this did not independently predict optimal use at multivariate analysis (p = 0.452).
		(*b*) Report category boundaries when continuous variables were categorized	8	A total of two hundred ninety-three (293) ALHIV with a median age of 15.0 years (IQR

			13.0–17.0) from the study settings participated in the study (Table 1).
	(*c*) If relevant, consider translating estimates of relative risk into absolute risk for a meaningful time period	N/A	

Continued on next page

Other analyses	17	Report other analyses done—eg analyses of subgroups and interactions, and sensitivity analyses	N/A	
**Discussion**				
Key results	18	Summarise key results with reference to study objectives	8-12	The findings in this study show that there is poor utilization of ART services among adolescents living with HIV in northern Uganda. As a result, of the expected 95% viral suppression rate(1) we found a viral suppression rate of only 63.1%.
Limitations	19	Discuss limitations of the study, taking into account sources of potential bias or imprecision. Discuss both direction and magnitude of any potential bias	15	The study was conducted in only three health facilities within Lira municipality and hence the findings may not necessarily be generalizable to the whole region or other contexts. However, the facilities selected are high-volume facilities with wide catchment areas, making the findings externally valid to a good extent. The use of Odd ratios (Ors) from logistic regression instead of the relative risk ratio from the Poisson regression carried the risk of overestimating the association between the predictors and the outcome.
Interpretation	20	Give a cautious overall interpretation of results considering objectives, limitations, multiplicity of analyses, results from similar studies, and other relevant evidence	13-15	According to our study, the best use of ART services was among younger adolescents. Adolescents aged 10 to 14 were thrice more likely than those aged 15 to 19 to

				optimally use ART services. An observation by Nachega et al. in South Africa that older adolescents living with HIV were less adherent and had lower rates of virologic suppression and immunological recovery(25) is supported by the subpar optimization of ART services among older adolescents in our study. Our finding also resonates with that of Mustapha et al, who found that only 19.7% of adolescent mothers aged 15 - 19 in Mulago Hospital, Uganda, optimally utilized the services for PMTCT(26). The apparent independence and lack of consideration shown to older teenagers in both family and community settings may be one explanation for this finding.
Generalisability	21	Discuss the generalisability (external validity) of the study results	15	The study was conducted in only three health facilities within Lira municipality and hence the findings may not necessarily be generalizable to the whole region or other contexts.
**Other information**				
Funding	22	Give the source of funding and the role of the funders for the present study and, if applicable, for the original study on which the present article is based	16	"Research reported in this publication was supported by the Fogarty International Center (the U.S, Department of State's Office

		of the U.S. Global AIDS Coordinator and Health Diplomacy [S/GAC] and the President's Emergency Plan for AIDS Relief [PEPFAR]) of the National Institutes of Health under Award Number R25TW011210. The content is solely the responsibility of the authors and does not necessarily represent the official views of the National Institutes of Health".

*Give information separately for cases and controls in case-control studies and, if applicable, for exposed and unexposed groups in cohort and cross-sectional studies.

Note: An Explanation and Elaboration article discusses each checklist item and gives methodological background and published examples of transparent reporting. The STROBE checklist is best used in conjunction with this article (freely available on the Web sites of PLoS Medicine at http://www.plosmedicine.org/, Annals of Internal Medicine at http://www.annals.org/, and Epidemiology at http://www.epidem.com/). Information on the STROBE Initiative is available at www.strobe-statement.org.
